# Evaluation of the Hypoglycemic Activity of *Morchella conica* by Targeting Protein Tyrosine Phosphatase 1B

**DOI:** 10.3389/fphar.2021.661803

**Published:** 2021-05-14

**Authors:** Naeema Begum, Abdul Nasir, Zahida Parveen, Taj Muhammad, Asma Ahmed, Saira Farman, Nargis Jamila, Mohib Shah, Noor Shad Bibi, Akif Khurshid, Zille Huma, Atif Ali Khan Khalil, Ashraf Albrakati, Gaber El-Saber Batiha

**Affiliations:** ^1^ Department of Biochemistry, Abdul Wali Khan University, Mardan, Pakistan; ^2^ Department of Molecular Science and Technology, Ajou University, Suwon, South Korea; ^3^ Institute of Molecular Biology and Biotechnology (IMBB), The University of Lahor, Lahor, Pakistan; ^4^ Department of Chemistry, Shaheed Benazir Women University of Science and Technology Peshawar, Peshawar, Pakistan; ^5^ Department of Botany, Abdul Wali Khan University, Mardan, Pakistan; ^6^ Department of Botany, University of Peshawar, Peshawar, Pakistan; ^7^ Department of Biological Sciences, National University of Medical Sciences, Rawalpindi, Pakistan; ^8^ Department of Human Anatomy, College of Medicine, Taif University, Taif, Saudi Arabia; ^9^ Department of Pharmacology and Therapeutics, Faculty of Veterinary Medicine, Damanhour University, Damanhour, Egypt

**Keywords:** protein tyrosine phosphatase 1B activity, hyperglycemia, diabetes, cytotoxicity, liquid chromatography–mass spectrometry, *Morchella conica* pers., streptozotocin

## Abstract

*Morchella conica* (*M. conica*) Pers. is one of six wild edible mushrooms that are widely used by Asian and European countries for their nutritional value. The present study assessed the anti-diabetic potential of *M. conica* methanolic extract (100 mg/kg body weight) on streptozotocin (STZ)-induced diabetic mice. STZ was used in a single dose of 65 mg/kg to establish diabetic models. Body weights, water/food intake and fasting blood glucose levels were measured. Histopathological analysis of the pancreas and liver were performed to evaluate STZ-induced tissue injuries. In addition, *in vitro* assays such as α-amylase and protein tyrosine phosphatase 1B (PTP1B) inhibitory, antiglycation, antioxidant and cytotoxicity were performed. The *in vitro* study indicated potent PTP1B inhibitory potential of *M. conica* with an IC_50_ value of 26.5 μg/ml as compared to the positive control, oleanolic acid (IC_50_ 36.2 μg/ml). *In vivo* investigation showed a gradual decrease in blood sugar level in *M. conica-*treated mice (132 mg/dl) at a concentration of 100 mg/kg as compared to diabetic mice (346 mg/dl). The extract positively improved liver and kidney damages as were shown by their serum glutamic pyruvic transaminase, serum glutamic oxaloacetate, alkaline phosphatase, serum creatinine and urea levels. Histopathological analysis revealed slight liver and pancreas improvement of mice treated with extract. Cytotoxicity assays displayed lower IC_50_ values. Based on the present results of the study, it may be inferred that *M. conica* are rich in bioactive compounds responsible for antidiabetic activity and this mushroom may be a potential source of antidiabetic drug. However, further studies are required in terms of isolation of bioactive compounds to validate the observed results.

## Introduction

Protein tyrosine phosphatase 1B (PTP1B) is a non-trans membranous protein that acts as a major negative regulator of insulin signaling pathways, thereby mediating insulin resistance (Haj et al., 2003; Cho, 2013; Abdelsalam et al., 2019; Hussain et al., 2019). The protein tyrosine phosphatases (PTPs) constitute a family of closely related key regulatory enzymes that dephosphorylate phosphotyrosine residues in their protein substrates (Barford, 1996; Alonso et al., 2004; Bialy and Waldmann, 2005). They provide the necessary biological counterpart to protein kinases in signal transduction pathways and play an important role in the regulation of many cellular processes such as cell growth and differentiation, metabolism, cell migration, immune response, cell apoptosis and bone development (Tonks, 2006; Hendriks et al., 2013). Malfunctions in PTP activity lead to aberrant tyrosine phosphorylation associated with various diseases such as diabetes, obesity, cancer, inflammation and neurodegenerative diseases (Bialy and Waldmann, 2005; Hendriks et al., 2013; Arthur et al., 2020). Therefore, the development of therapeutically promising potent PTP inhibitors is of great importance. Inhibition of PTP1B alleviates the resistant state by removing negative pressure on the pathway (Sun et al., 2016). Due to its dual specificity, PTP1B is an effective target for the treatment of both type 2 diabetes (T2DM) and obesity (Ha et al., 2020; Hsing et al., 2020; Nandi and Saxena, 2020). The development of potent and selective PTP1B inhibitors that engage both positively charged active-site pocket and highly conserved non-catalytic sites, is challenging. Several strategies are being pursued to improve the pharmacological properties of PTP1B inhibitors, specifically small-molecule therapeutics with the requisite potency and selectivity into orally available drugs with desirable physicochemical properties and *in vivo* efficacy (Shinde et al., 2018). A phenolic compound, ferulic acid commonly found in a variety of plants, restored insulin signaling alteration and PTP1B regulation in experimental diabetic rats fed with a high fructose diet (Wang et al., 2017).

Several studies reported strong correlation between PTP1B expression and insulin resistant state (Choi and Kim, 2010; Panzhinskiy et al., 2013; Zhou et al., 2021). Insulin-stimulated glucose disposal has been significantly improved in PTP1B deficient mice, thus reducing overexpression of PTP1B may enhance the insulin signaling pathway (Klaman et al., 2000). Although several studies are available that explain both *in vitro* and *in vivo* inhibition of PTP1B, there is still not any single promising inhibitor available from natural source for PTP1B (Li et al., 2019; Lei et al., 2020). In addition, limited literature is available on pancreatic PTP1B expression analysis with reference to molecules that may also control the hyperglycemic index and altered expression of PTP1B protein in liver (Waring et al., 2003; Owen et al., 2013; Figueiredo et al., 2019). It has been noted in the literature that overexpression of PTP1B protein in the liver tissue leads to permanent switch-off of insulin signaling and therefore increase hyperglycemic index due to insulin resistance mechanism (Zabolotny et al., 2004). Secondly, pancreatic PTP1B deficiency leads to decreased insulin secretion through ER stress mechanism. Therefore, the modulation of the expression of PTP1B in the liver and/or pancreas can be a potential target for drug treatment of diabetes (Choi and Kim, 2010; Panzhinskiy et al., 2013; Mobasher et al., 2014). Therefore, the present study was conducted to evaluate PTP1B modulation *in vitro* and *in vivo* by using extracts of *M. conica*. In addition, liver and pancreatic PTP1B expression profiling was conducted to understand the underlying mechanism of insulin resistance. LC-MS analysis was also carried out to assess the compound profile in *M. conica.*

## Methods and Materials

### Sample Preparation

Fresh samples of *M. conica* were purchased from a local market and authenticated by Dr Mohib Shah, Assistant Professor, Department of Botany. The specimens were deposited in the herbarium Department of Botany, Abdul Wali Khan University, Mardan. Shed dried whole plant was ground to a fine powder and soaked in methanol (1:10 w/v) for three consecutive days. Samples were then filtered, centrifuged, and residues were collected for the second round of extraction. The extraction process was done thrice and extracts stored at 4°C for further analysis.

### *In vitro* Assays

#### Protein Tyrosine Phosphatase 1B Inhibition Assay

PTP1B inhibition by crude methanol extract of *M. conica* was carried out using a slight modification to the method earlier described (Cui et al., 2006). Briefly, PTP1B (100 µl of 0.5 μg/ml stock solution) in 50 mM citrate buffer (pH 6.0) containing 0.1 M NaCl, 1 mM dithiothreitol (DTT) and 1 mM ethylenediaminetetraacetic acid (EDTA) was added to each well of a 96-well plastic plate. Next, sample (2.0 µl in DMSO) was added to each well and incubated for 10 min at 37°C. The reaction was initiated by the addition of *p*-NPP (100 µl of 4.0 mM stock solution) in the citrate buffer, incubated at 37°C for 30 min and then terminated with the addition of 10 µl stop solution (10 M NaOH). The optical density of each well was measured at 405 nm using microplate reader ELISA (HER 480 HT). PTP1B inhibitory activity (%) is defined as

$$\left[1-\frac{\left({\text{ABS}}_{\text{sample}}-{\text{ABS}}_{\text{blank}}\right)}{\left({\text{ABS}}_{\text{control}}-{\text{ABS}}_{\text{blank}}\right)}\right]\times 100$$

Where ABS_blank_ is the absorbance buffer and *p*-NPP, ABS_control_ is the absorbance of *p*-nitrophenol, and ABS_sample_ is that with a test sample. All assays were performed in triplicate. Oleanolic acid, a known phosphatase inhibitor, was used as a positive control.

#### α-Amylase Inhibition Assay

Screening of crude methanol extract was carried out with slight modifications to the method earlier published (Xiao et al., 2006). The total reaction volume (3 ml) composed of 120 µl of sodium phosphate buffer (0.02 M) at pH 6.9 (containing 6 mm sodium chloride), 1.5 ml pancreatic α-amylase solution (0.05 mg/2 ml H_2_0) and plant extracts of 400 µl at concentration of 30–1000 μg/ml. The reaction mixture was incubated at 37°C for 10 min, followed by the addition of soluble starch (1%, w/v). The mixture was incubated again at 37°C for 15 min. The enzymatic reaction was stopped by the addition of 1 M HCl (60 µl). Finally, a 300 µl of iodine reagent (5 mM I_2_ and 5 mM KI) was added. The color change was noted at the absorbance of 620 nm on a spectrophotometer (721 2C50811136 Shimadzu, Japan). The control reaction representing 100% of enzyme activity did not contain any plant extract. To eliminate the absorbance produced by plant extract, appropriate extract controls without the enzyme were also included. The known amylase inhibitor, acarbose, was used as a positive control at a concentration range of 6.5–32.8 μg/ml. A dark-blue color indicated the presence of starch; yellow color indicated the absence of starch while a brownish color indicated partially degraded starch in the reaction mixture. In the presence of inhibitors from the extracts, the starch added to the enzyme assay mixture was not degraded, resulting in a dark-blue color.

#### Antiglycation Assay

Antiglycation assay was conducted according to the reported methods with slight modification (Nakagawa et al., 2002). In all experiments, the final reaction volume was 1200 µl that comprised of 400 µl bovine serum albumin (BSA) (10 mg/ml), 400 µl of glucose anhydrous (50 mg/ml) and 400 µl test sample. Glycated control contained 400 μl BSA, 400 µl glucose and 400 µl sodium phosphate buffer, while blank control contained 400 µl BSA and 800 µl sodium phosphate buffer. The reaction mixture was incubated at 37°C for 7 days. After incubation, 120 µl of trichloroacetic acid (TCA) was added and centrifuged (15,000 rpm) for 4 min at 4°C. After centrifugation, the pellets were rewashed with 1200 µl (10%) of TCA. The supernatant containing glucose, inhibitor and interfering substances were removed while pellets containing advance glycated end products (AGE)-BSA were dissolved in 1200 µl phosphate buffer solution (PBS). Assessment of fluorescence spectrum (excitation 370 nm) and changes in fluorescence intensity (excitation 370 nm to emission 440 nm) based on AGEs were monitored by using spectrofluorophotometer (RF-5301PC, Shimadzu, Japan).

#### 2,2-Diphenyl-1-Picrylhydrazyl Hydrate Radical Scavenging Assay

The antioxidant activity of plant extracts against stable 2,2-diphenyl-1-picrylhydrazyl hydrate (DPPH) was conducted according to the procedure reported with slight modifications (Brand-Williams et al., 1995). Briefly, the solution contained 1 ml of the methanolic extract of the plant at a concentration range of 30–1000 μg/ml and 2 ml of 0.1 mM DPPH solution. A standard solution of L-Ascorbic acid (1–100 μg/ml) was prepared. About 1 ml of methanol with 2 ml of DPPH solution was prepared for negative control. The samples were vortexed and kept in dark for 5 min at room temperature and then the decrease in absorbance at λ = 517 nm was recorded against a control without crude extract. The dose-response curve was generated to calculate IC_50_ values. The experiment was carried out in triplicates.

#### Cytotoxicity Assay Using Thiazolyl Blue Tetrazolium Bromide

The resistant CCRF-CEM/VCR-1000 cell line was received from Prof. Dr. Volker Gekeler (Gekeler et al., 1992) and maintained in RPMI 1640 medium containing 10% Fetal Calf Serum (FCS) and 1000 ng/ml vincristine. Thiazolyl blue tetrazolium bromide (MTT) assay was used to determine cell proliferation as described (Zhang et al., 2012). CCRF-CEM/VCR-1000 cells were seeded at a density of 10,000 cells per well in a 96-well plate. Different concentrations of *M. conica* was added to these wells and incubated overnight at 37°C under 5% CO_2_. The MTT solution (3 mg/ml) was added into the media and incubated for 4–6 h. Deep Purple color of formazan appeared in the wells, which was dissolved in 200 µl of a solubilising agent or dimethyl sulfoxide (DMSO). Absorbance was read at 540 nm and the background was subtracted at 670 nm. CCRF-CEM/VCR-1000 cells were counted by an automated cell counter (AMQAX1000 Countess TM II Automated Cell Counter, Thermo Fischer Scientific).

### *In vivo* Anti-Diabetic Activity

#### Experimental Design and Procedure

The experimental animals; adult male BALB/C mice (average weight 25–36 g) were obtained and housed at Veterinary Research Institute, Peshawar, KPK, Pakistan. The animals were acclimatized in steel cages for a week (12 h light/dark cycle, 27 ± 2°C) and were provided with standard food pellet and water. A total number of 24 mice were divided into four groups (*n* = 6 mice/group).

Group 1: Normal control mice, received normal chaw food and water.
Group 2: Diabetic control mice, received normal chaw food and water but no treatment.
Group 3: Diabetic mice received normal chaw food and water and treated with glibenclamide (0.18 mg/kg of body weight).
Group 4: Diabetic mice received normal chaw food and water and treated with *M. conica* (100 mg/kg).

#### Streptozotocin Induced Diabetes Mellitus Type II

Streptozotocin (STZ) was dissolved in a freshly prepared cold citrate buffer (pH 4.5) and administered intraperitoneally 65 mg/kg body weight. After 48 h of induction, glucose level was checked and mice with glucose level above 200 mg/dl were considered diabetic. Food was removed from mice 4–6 h before induction. The glucose level of normal mice was checked (normal range 75–150 mg/dl).

#### Treatment Administration

The methanol extract of *M. conica* was dissolved in 5% Tween 80 at a final concentration of 100 mg/kg and treatments were given once daily intraperitoneally for 4 weeks. Blood was sampled from the tail vein and fasting blood glucose was measured using a portable glucometer (Accu-Chek, Roche, Germany) at 1 week interval for four weeks. At the end of the experiment, all animals were fasted overnight and anesthetized with chloroform. The blood collected was centrifuged at 4000×*g* for 15 min at 30°C, for biochemical analysis by using Hitachi 902 Automatic Chemical Analyser kit (Japan). The mice were sacrificed by cervical dislocation and liver and pancreas were carefully excised, rinsed in ice-cold saline, dehydrated in gradual ethanol (50–100%), cleared in xylene and then embedded in paraffin wax for histopathological studies. Both liver and pancreas were stained using Hematoxilin and Eosin (H and E) stain (Juárez-Rojop et al., 2012).

### Expression Analysis of Protein Tyrosine Phosphatase 1B

For expression analysis, a simple step ELISA kit was used according to manufacturer’s instruction (ab184865, Abcam). A total of 200 mg of the sampled liver stored in 10% formalin was minced thoroughly, rinsed in PBS and then homogenized in 500 μl cell extraction buffer PTR to prepare tissue lysates. After incubating in ice for 20 min, centrifuged at 18,000×*g* for 20 min at 4°C. The supernatant was transferred to a clean tube. About 50 µl of samples and standard were added to the wells. Next, 50 µl of antibody cocktail was added to each well. The sealed plate was then incubated for 1 h on a shaker set to 400 rpm at room temperature. Washed each well with wash buffer PT. A 100 µl of TMB substrate was added to each well and incubated for 10 min in the dark on a shaker set to 400 rpm. A total of 100 µl of stop solution was added to each well and shaken thoroughly for 1 min. Finally, absorbance was recorded at 450 nm.

### Liquid Chromatography–Mass Spectrometry Analysis Method

The chromatographic separations for the determination of molecular masses of major phytochemicals were performed on UPLC (Shimadzu, Kyoto, Japan) coupled with ion trap-time of flight (IT-TOF) mass spectrometer using positive as well as negative ESI mode at 4500 V. The separation column used was Capcell Core C18 column (2.1 mm × I.D. x 150 mm, 2.7 µm, Shiseido, Japan). The mobile phase consisted of (A) HPLC grade water (0.1% formic acid) and (B) methanol (0.1% formic acid). A gradient program time was 18 min at the flow rate of 5 μL/min. The detector, diode array was set at 190–600 nm. At the end of each run, 100% B was allowed to flush the column for 10 min. For mass detection, the samples were scanned over *m*/*z* 100–1,000. All data was processed by Bruker Compass Data Analysis 4.2 software and an accurate mass calculator. The spectral peaks in the MS spectra of the subject samples were identified by comparing them with the LC-MS database Willey 8, NIST library.

### Statistical Analysis

The obtained data was analyzed using non-linear regression analysis in GraphPad Prism 5 and the test of significance was done using one-way ANOVA by applying Tukey’s test. IC_50_ values were calculated from the dose-response curve. All the values were expressed as mean ± standard error of the mean (S.E.M). Differences between groups were considered significant at *p* < 0.05 and *p* < 0.001 levels.

## Results

### *In vitro* Protein Tyrosine Phosphatase 1B Inhibition

**FIGURE 1:**
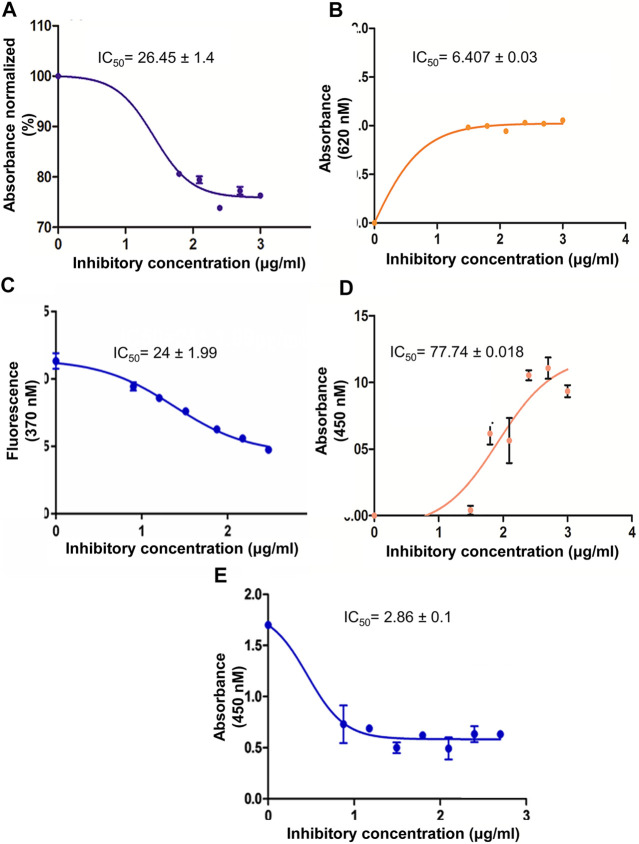
**(A)** Dose response curve for PTP1B inhibition activity by *M. conica*. PTP1B inhibition activity expressed as a percentage of control (100%) by *M. conica*. **(B)** The hyperbolic dose response curves of α-amylase inhibition activity of methanol extract *M. conica.* The figure demonstrates that an increase in absorbance results in an increase in inhibition. **(C)** The hyperbolic dose response curves of antiglycation activity of methanol extract *M. conica*. The figure display that decrease fluorescence corresponds to increased inhibition. **(D)** DPPH radical scavenging activity of methanol extract of *M. conica*. **(E)** A dose response curve of cytotoxicity of *M. conica* against ABCB1 overexpressing cell line using MTT assay. The results are presented as the mean ± SEM of three independent experiments, each of which was carried out in triplicate. IC_50_ values were calculated from the curves.

In an initial screen, the crude methanolic extract of *M. conica* was evaluated in an *in vitro* PTP1B inhibitory assay. A concentration range of 30–1000 μg/ml was assessed. The *M. conica* extract showed 80% inhibition in PTP1B activity with an IC_50_ value of 26.5 ± 1.48 μg/ml, significantly lower than that of positive control, IC_50_ 36.2 ± 2.42 μg/ml (Figure 1).

### α-Amylase Inhibition

The crude methanolic extract was evaluated at a concentration range of 30–1000 μg/ml. The results are shown in Figures 1A,B. The dose-response curve for methanolic extract was constructed and the percent α-amylase inhibition and the IC_50_ values were determined from the dose-response calibration curve. The activity of the extracts was concentration-dependent.

### Antiglycation Activity

The antiglycation activity of *M. conica* was evaluated using different concentrations. Results are shown in Figure 1C. The activity was also monitored in the absence of an inhibitor as negative control and the presence of rutin as a positive control. Data indicated that *M. conica* showed a positive correlation of activity with concentration. Moreover, the IC_50_ value was found to be 24 ± 1.99 μg/ml. The comparison was made with rutin, which is a known reported antiglycation compound that showed an IC_50_ value of 18 ± 0.98 μg/ml.

### Antioxidant Activity

The antioxidant activity of *M. conica* was evaluated using different concentrations of methanolic extract (Figure 1D). The study was controlled using positive and negative controls. In this regard, ascorbic acid was used as standard and a reaction mixture in the absence of an inhibitor was used as negative control. Dose response curves indicated a trend in the activity. The data was compared based on IC_50_ value which was found to be 77.74 ± 0.018 μg/ml for *M. conica*. The low IC_50_ value of *M. conica* suggests that it possesses high oxidative stress inhibitory potential.

### Cytotoxicity Assay Using Thiazolyl Blue Tetrazolium Bromide

Results of cytotoxicity assay revealed potent inhibition of CCRF-CEM/VCR-1000 overexpressing cell proliferation showing IC_50_ values of 2.862 ± 0.113 μg/ml (Figure 1E). The data demonstrated higher potency of *M. conica* against PTP1B and was thus further evaluated in the *in vivo* STZ-induced diabetic mice model for its antidiabetic potential and its effect on protein tyrosine phosphatase 1B expression.

### *In vivo* Study

#### Effect of *M. conica* on Body Weight and Hyperglycemic Index

**FIGURE 2:**
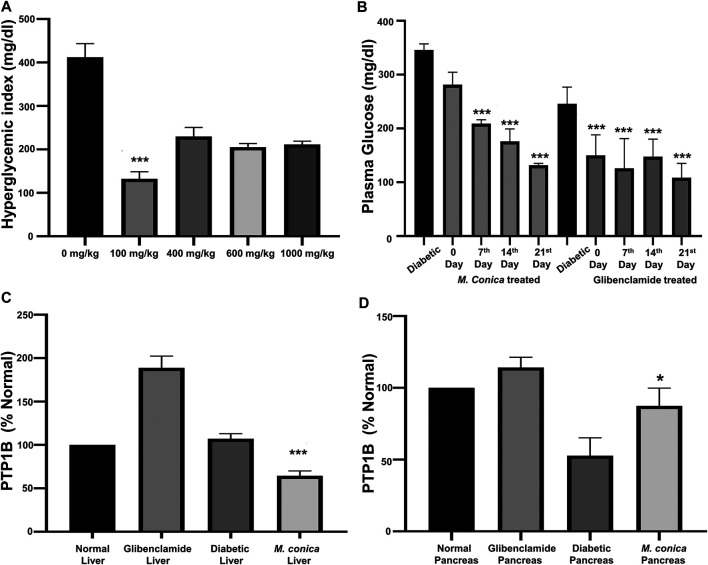
**(A)** Dose optimization for *M. conica* to select a final effective dose to be administered which was found to be 100 mg/kg. At this dose the sample showed significant results at ***p < 0.001. **(B)** Effect of *M. conica* on hyperglycemic index compared with glibenclamide treated group. The results are presented as the mean ± SEM of three independent experiments, each of which was carried out in triplicate. The data was analyzed using one-way ANOVA by applying Tuckey test. For *M. conica* results were found to be significant (***p < 0.001) from 14 days onward. **(C)** Comparison of PTP1B expression in liver tissue ***p = 0.001. **(D)** Comparison of PTP1B expression in pancreas tissue (*p = 0.16) of normal mice, diabetic mice and diabetic treated groups with glibenclamide and *M. conica*. The results are presented as the mean ± SEM of three independent experiments, each of which was carried out in triplicate.

The body weight of animals from all groups including normal, diabetic control, diabetic treated with glibenclamide, and *M. conica* were recorded. In this section body weights of animals (*n =* 6) treated with *M. conica* were evaluated. It had been noticed that under diabetic conditions, the body weight was decreased with compared with normal mice. The values were found to be 26.9 ± 4.52 g and 34 ± 3.59 g for the diabetic and normal groups, respectively. For glibenclamide and *M. conica*, treated groups the average body weights were found to be 33.1 ± 1.82 g and 32.6 ± 3.42 g, respectively. The methanol extract of *M. conica* was also evaluated for its effect on the hyperglycemic index of the treated group (Figure 2A). Dose-response curve was initially generated to optimize the dose. Four different concentrations were given to diabetic mice including 100, 400, 600 and 1000 mg/kg. It had been noticed that the *M. conica* extract controlled the hyperglycemic index from 448 to 148 mg/dl at a concentration of 100 mg/kg. Therefore, this dose has been selected for further analysis. The treated group was administered 100 mg/kg of *M. conica* extract intraperitoneally for up to 4 weeks. The hyperglycemic index was recorded on weekly basis for up to 4 weeks for normal mice, diabetic mice, and diabetic mice treated with *M. conica* (Figure 2B). At zero day, the hyperglycemic index was recorded as 346 ± 11.5 mg/dl. Results revealed the drop in glycemic index to 281 ± 23.2 mg/dl for *M. conica* and 216 ± 37.8 mg/dl for glibenclamide treated group after first week. The glycemic index was gradually decreased to 132 ± 3.51 mg/dl and 110 ± 26.6 mg/dl after the fourth week for *M. conica* and glibenclamide treated groups, respectively.

#### Effect of *M. conica* on Lipid Profile of Treated Mice

After the fourth week, all mice were euthanized and organs were collected for histopathological analysis. Blood was collected for serum chemistry analysis including total cholesterol, triglycerides, high-density lipoprotein cholesterol (HDL), low-density lipoprotein cholesterol (LDL), serum creatinine, and urea. Results of the *in vivo* study revealed an increase in total cholesterol in diabetic mice (198 mg/dl) as compared to normal mice (108 mg/dl) where HDL level was 28 mg/dl and LDL was 80 mg/dl for a normal group (Table 1). However, in the diabetic group, HDL was found to be 93 mg/dl and LDL value was 105 mg/dl. In case of the *M. conica*-treated group, the values for total cholesterol was found to be 141.5 ± 6.36 mg/dl. The HDL and LDL values were found to be 50 ± 1.41 mg/dl and 91.5 ± 4.95 mg/dl, respectively. When the triglycerides profile was compared, an increased level of triglycerides in diabetic mice (190 mg/dl) was noticed as compared to normal (116 mg/dl). The *M. conica-*treated group possessed a triglyceride level of 187.6 ± 3.67 mg/dl and for the positive control glibenclamide treated group triglycerides were found to be 188 ± 3.87 mg/dl.

**TABLE 1:** Levels of TG, TC, HDL and LDL in the normal, diabetic control, and glibenclamide and *M*. *conica*-treated mice group. Values are means of triplicate determination (*n* = 3) ± standard deviation.

Sample	Body weight	TG (mg/dl)	TC (mg/dl)	HDL (mg/dl)	LDL (mg/dl)
Diabetic control	33.84 ± 3.59	190 ± 3.25	198 ± 5.01	93 ± 4.31	105 ± 5.09
Normal	26.9 ± 4.52	116 ± 4.63	108 ± 4.13	80 ± 3.90	28 ± 5.91
Glibenclamide treated	33.13 ± 1.82	188 ± 3.87	139 ± 3.51	78 ± 2.39	120 ± 4.08
*M*. *conica-*treated	32.6 ± 3.42	187.6 ± 3.67	141.5 ± 6.36	50 ± 1.41	91 ± 4.95

TG, triglyceride; TC, total cholesterol; HDL, high-density lipoprotein cholesterol; LDL, low-density lipoprotein cholesterol.

#### Effect of *M. conica* on Liver and Kidney Damage

Diabetes leads to liver and kidney damage therefore the effect of *M. conica* was also evaluated on liver and kidney. Both SGP and SGO levels were increased in the bloodstream of diabetic mice. The values were found to be 77 U/l and 79 U/l as compared to normal mice having SGP and SGO values of 45 U/l and 44 U/l respectively. When the diabetic treated group with *M. conica* was evaluated it had been noticed that the SGP and SGO levels were found to be 65.5 ± 3.53 mg/dl and 58.5 ± 3.53 mg/dl respectively which were slightly less than that of a diabetic group. For glibenclamide treated groups SGP and SGO were 50 U/l and 59 U/l respectively. The when the alkaline phosphatase levels were compared among different groups, it had been notice that dibatec group have higher ALP level (268 U/l) as compared to normal control (98 U/l) thus indicated that in the diabetic group there is a damage in either liver, kidney or pancreas that leads to an elevated level of ALP. However, *M. conica*-treated group showed a slightly lower ALP level as compared to the diabetic group (225 U/l). In addition, serum urea and creatinine level were monitored for different groups. The data indicated that the diabetic group displays a higher level of serum urea (70 mg/dl) and creatinine level (1.7 mg/dl). However, in *M. conica*-treated group, the values were recorded as 48 ± 2.82 mg/dl and 0.30 ± 0.07 mg/dl for serum urea and creatinine, respectively, which indicated that *M. conica* reduced serum urea and creatinine to approximately normal level (Serum urea 33 ± 3.13 and creatinine 0.8 ± 0.06).

### Effect of *M. conica* on Protein Tyrosine Phosphatase 1B Expression in Liver and Pancreas

Under the diabetic conditions, PTP1B may become overexpressed in the liver, therefore, it was important to assess the effect of *M. conica* extract on the expression of PTP1B in the liver and pancreas. From the results (Figure 2C), it was found that in the diabetic liver, protein expression was almost doubled (188.7 ± 19.3%) to that of normal, which was taken as 100%. For the *M. conica*-treated group, the percent protein was found to be 64.9 ± 7.35. The treated group was also evaluated for pancreatic PTP1B expression. There was a decrease in the pancreatic PTP1B expression in the diabetic control group compared to the normal mice (Figure 2D). The STZ-induced diabetic group had a protein expression of 52.75 ± 17.6%. However, PTP1B expression was restored in the pancreas of glibenclamide treated group and *M. conica-*treated group with a percentage of 87.4 ± 12.4% and 64.9 ± 7.35, respectively.

### Histopathological Analysis for Liver, Pancreas and Kidney

**FIGURE 3:**
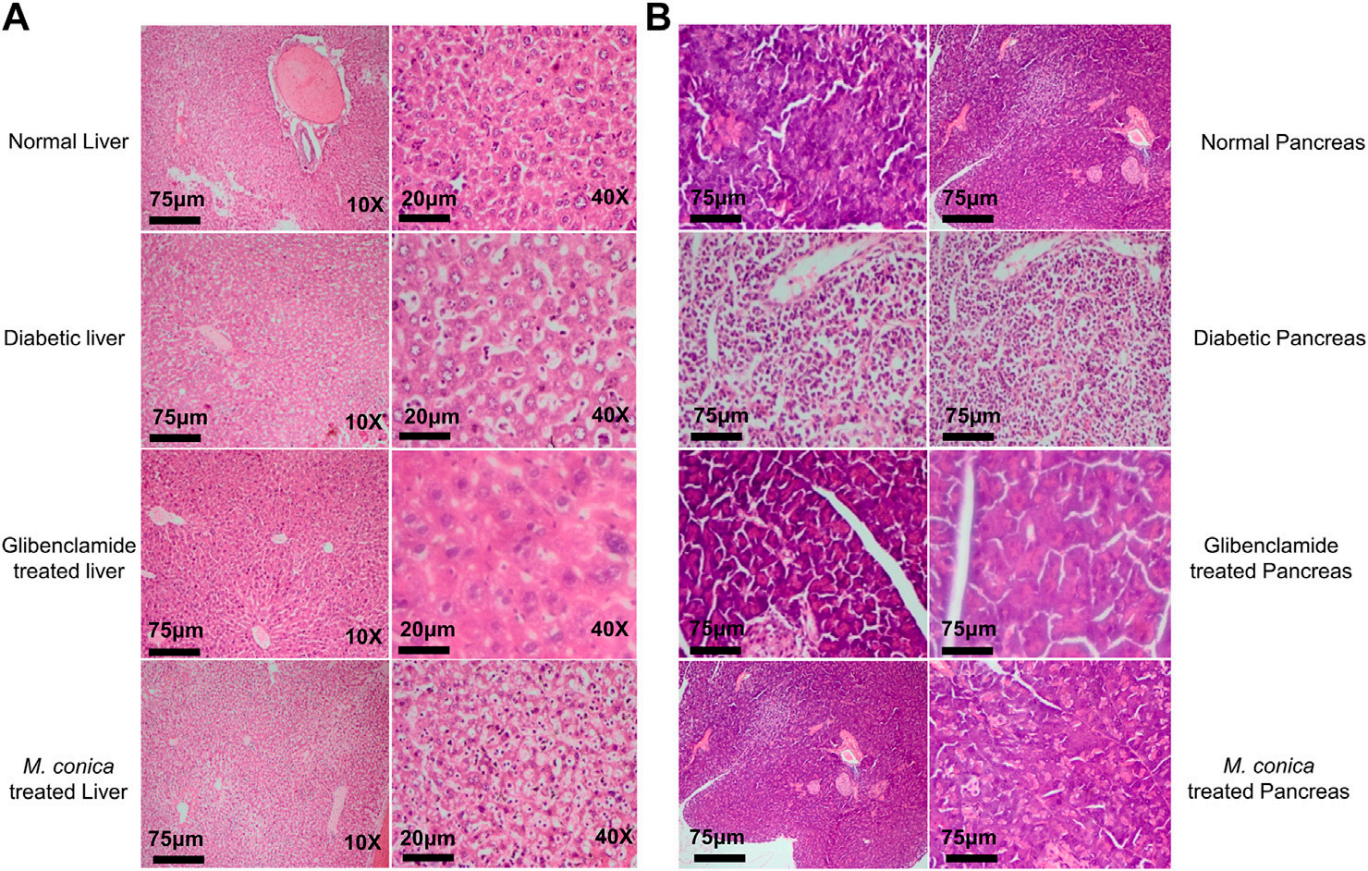
**(A)** *M. conica* restored the morphological integrity of the liver, as shown by histological examination. Liver tissue was stained with H and E (magnification, 10× scale bar 75 μm and 40× scale bar 20 μm). **(B)** *M. conica* restored the morphological integrity of the pancreas, as shown by histological examination. Pancrease tissue was stained with H and E (magnification, 10× scale bar 75 μm).

In the final series of analysis, collected organs including liver and pancreas from normal, diabetic, and treated groups were sectioned for histopathology. In the normal group, normal histology of sections of the liver including central vein, blood sinuses, hepatic cells and kuffer cells were observed (Figure 3A). Noticeable necrotic changes were present with severe degenerated parenchymal cells observed along with significant infiltration and inflammation of the hepatic and Kupffer cells. Deformed liver histology was due to inflammation. Shape changes were also seen in hepatocytes, central vein with dilation of sunsidus. While in the case of *M. conica*-treated groups minimal necrotic changes were observed in the liver as compared to diabetic control and glibenclamide treated groups. Parenchymal cells were also restored. Most interestingly hepatic cells were also found similar to normal control groups.

Histopathological analysis for the pancreas of normal group, diabetic group, glibenclamide and *M. conica*-treated groups are shown in Figure 3B. Results revealed that normal mice have 2 islets per X10 field. Histopathologic and necrotic changes were observed in the pancreas of diabetic mice. Severe distortion of exocrine cells and β-cells along with degenerative changes in β-cells were observed. The numbers of islet cells per X10 field were found to be 04. Treated groups with *M. conica* showed recovery and regeneration of cells of the pancreas with minimal necrotic changes and restoration of the normal cellular population with an abundant number of β-cells. Pancreatic tissues of treated mice for 29 days showed significantly reduced necrosis.

### Phytochemical Profiling of *M. conica* by Liquid Chromatography–Mass Spectrometry Analysis

**FIGURE 4:**
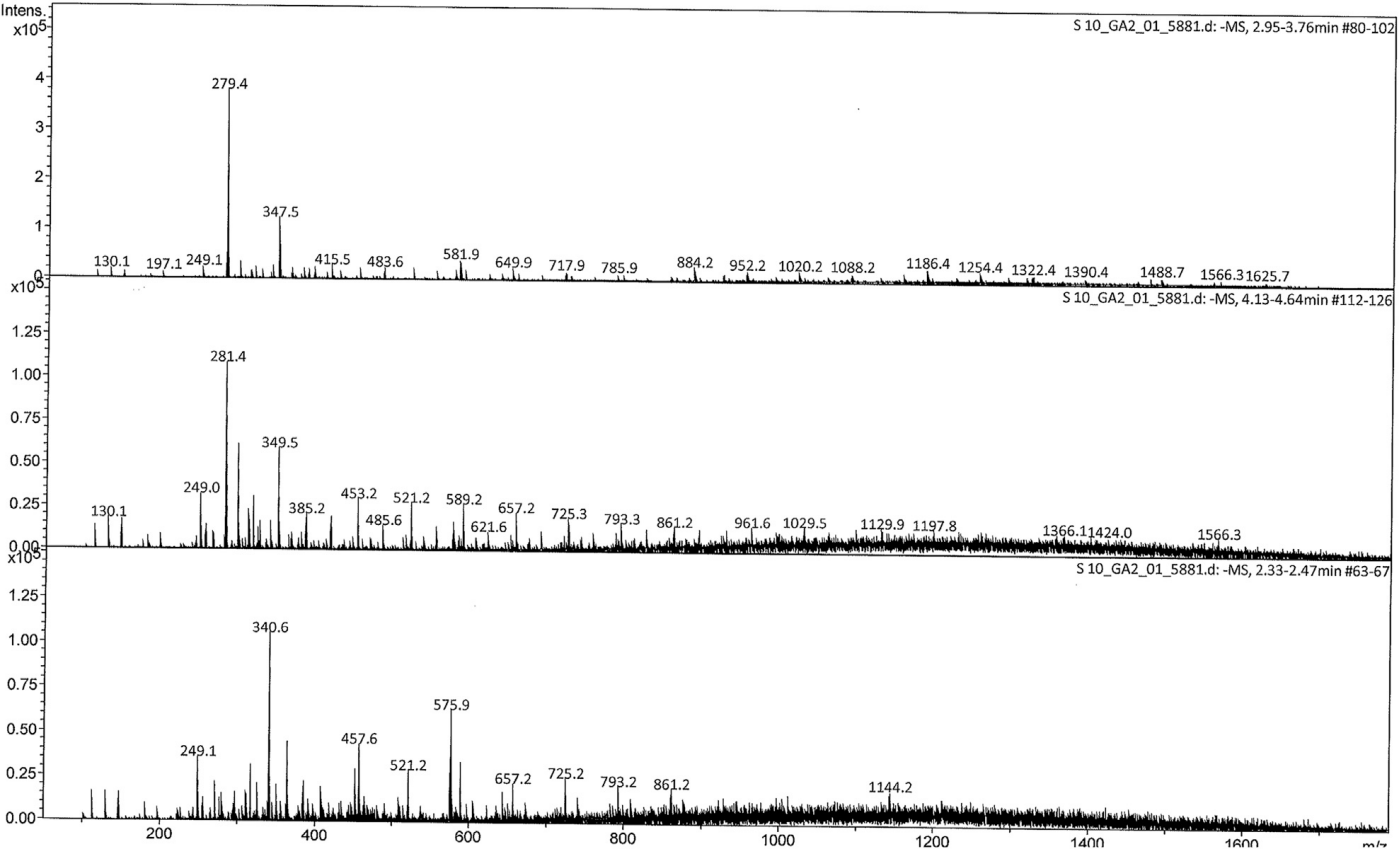
ESI mass spectrum (-MS) of *M. conica* at 2.95–3.76, 4.13–4.64, and 2.33–2.47 min full scan (200–1800 m*/z*).

**FIGURE 5:**
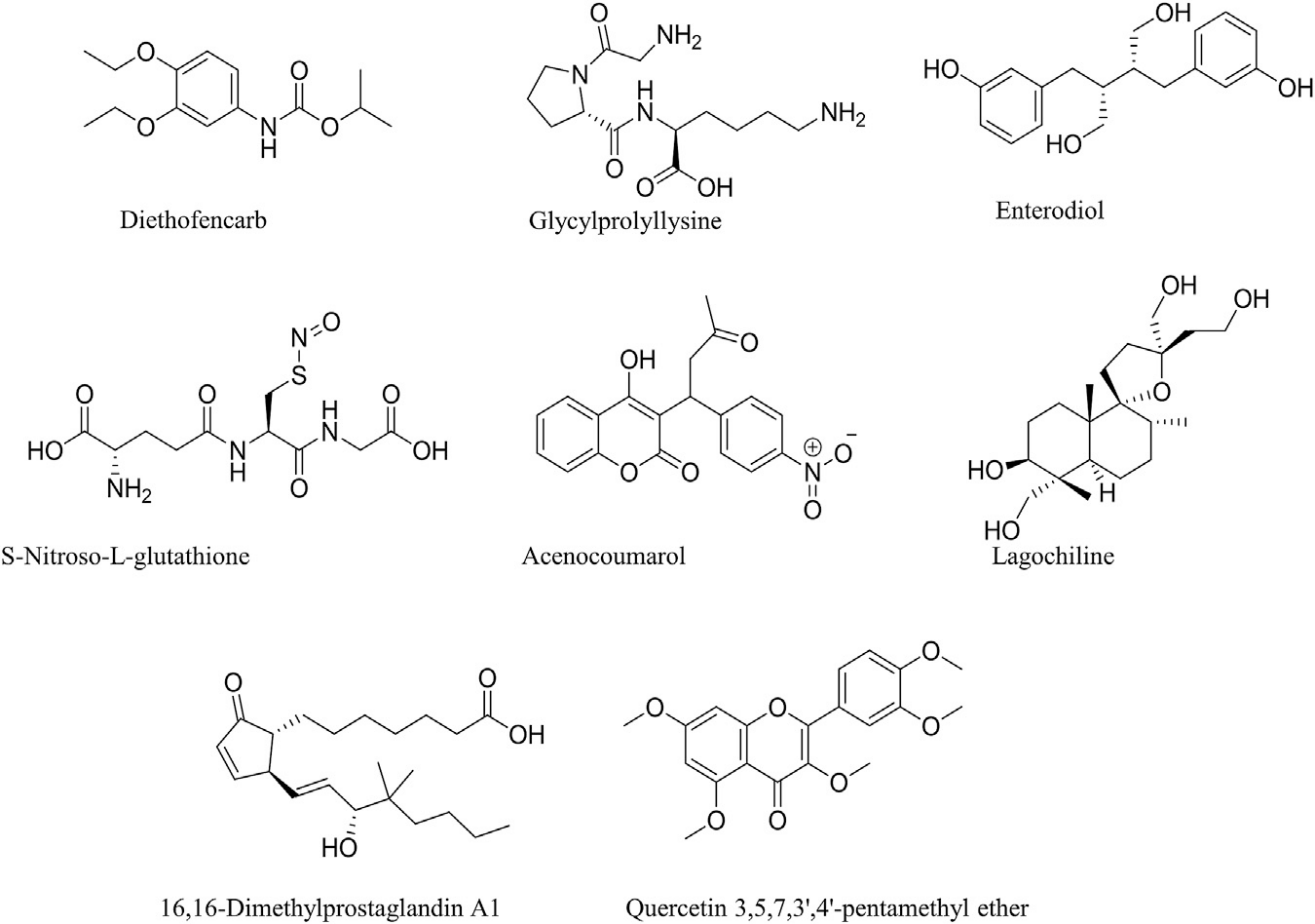
Chemical structures of identified Compounds in *M. conica*.

*M. conica* methanolic extract was analyzed by liquid chromatography-mass spectrometry (LC-MS) to identify possible compounds responsible for the observed antidiabetic activities. Regarding the LC-MS analysis, there is no previous report available on this investigation of *M. conica*. The LC chromatogram and the chemical structures of the compounds are given in Figures 4, 5 respectively, whereas its ESI-MS spectra in positive and negative mode are provided in the Supplementary Figure S1. Some of the compounds in the mass spectra obtained for *M. conica* were identified with the help of Willey 8, NIST library. The compounds are given in Table 2.

**TABLE 2:** Compounds in *M.conica* identified by NIST library.

S. No.	Compounds	Molecular weight	Molecular ion peak	Fragment ion peak	Spectrum type
1	Diethofencarb	267	289	226	[M+Na]^+^
2	Glycylprolyllysine	300	301	—	[M+H]^+^
3	Enterodiol	302	303	—	[M+H]^+^
4	S-Nitroso-L-glutathione	336	359	—	[M+Na]^+^
5	Acenocoumarol	353	352	—	[M−H]
6	Lagochiline	356	357	—	[M+H]^+^
7	16,16-Dimethylprostaglandin A1	364	365	—	[M+H]^+^
8	Quercetin 3,5,7,3′,4′-pentamethyl ether	372	373	—	[M+H]^+^

## Discussion

A recent known molecular target to treat diabetes is PTP1B which plays a significant role in the negative feedback of insulin signaling. Recently, various synthetic PTP1B inhibitors with sub-micromolar scale or nanomolar actions have been discovered by high-throughput screening and structure-based design (Combs et al., 2006; Combs, 2009). These compounds even though have great potential but yet experience limitation of the low cell permeability and low bioavailability which distressed their development as active candidate drugs (Lee and Wang, 2007; Zhang and Zhang, 2007). The real explanation for this could be the vicinity of very adversely negatively charged residue (containing difluoromethylphosphonates, carboxymethylsalicylic acids and oxalyl aminobenzoic acids) which mimic the phosphate group in insulin receptor substrate-1 (IRS-1) (Combs et al., 2006; Combs, 2009). Most of the work has been done with small molecular weight compounds to inhibit PTP1B. However, to date, none of these small molecular weight compounds are found to be successful in clinical trials and also in *in vivo* studies. Therefore, there is still a need for new inhibitors of PTP1B. In this regard, natural products can be used with potential sources of anti-diabetic drugs that target PTP1B overexpression.

In the present study, *M. conica* has been assessed in PTP1B inhibition both *in vitro* and *in vivo*. The *in vitro* data reveals the potency of *M. conica* against PTP1B by showing a percentage inhibition range of 73–80% with IC_50_ of 26.5 ± 1.48 μg/ml as compared to oleanolic acid with IC_50_ of 36.2 ± 2.42 μg/ml. Moreover, the *in vivo* data revealed that *M. conica* controlled the hyperglycemic index to normal levels at a dose of 100 mg/kg. PTP1B expression was found to be downregulated in the liver of the treated group. However, in the pancreas, there seemed an upregulation of PTP1B as compared to diabetic mice. In literature, role of the liver, PTP1B has been extensively studied and a detailed mechanism is known, however in the case of pancreatic PTP1B still very limited studies are available explaining its proper mechanism of action. Whole-body PTP1B knockout (KO) mice exhibit enhanced glucose tolerance and improved insulin sensitivity (Xi et al., 2015). Tissue-specific PTP1B deletion helped to define the functions of this phosphatase in many tissues including muscle, liver, and brain. In addition, several studies also reported the involvement of PTP1B in pancreas function. A compound knock out mouse model demonstrated that PTP1B global deficiency decreased severe diabetes caused by insulin receptor substrate 2 deletions. Mice with pancreatic PTP1B deficiency leads to impaired glucose tolerance and improper glucose stimulated insulin secretion (GSIS) (Dsouza and Lakshmidevi, 2015). Wang et al., 2017 reported the antidiabetic potential of *Ganoderma lucidum*. The group previously reported Fudan-Yueyang-*Ganoderma lucidum* (FYGL) from *G. lucidum* as a novel PTP1B inhibitor with an IC_50_ value of 5.12 mg/ml. It had been noticed that oral administration of FYGL for 4 weeks significantly decreased plasma glucose in STZ induced diabetic mice. FYGL also controlled the biochemistry indices relative to T2DM-accompanied lipidaemic disorders. It was concluded that the decrease in plasma glucose was due to the inhibition of PTP1B expression and activity. Our results were found consistent with this study as *M. conica,* an edible mushroom, controlled hyperglycemic index as well as PTP1B expression. In STZ-induced diabetic mice PTP1B protein was overexpressed and in diabetic mice treated with *M. conica* protein expression was decreased to normal levels. Therefore, it might be possible that the decrease in a hyperglycemic index is due to the inhibition of liver PTP1B.

Very recently PTP1B inhibition was studied using methanol extract of the fruit of *Paulownia tomentosa*. The extract showed potent inhibition of both PTP1B and glucosidase. The isolated eight different flavonoids from the extract with IC_50_ for PTP1B were found in the range of 1.9–8.2 µM. Most of the compounds were highly effective against PTP1B than α-glucosidase (Song et al., 2017). *M. conica* evaluated in the current study also showed potent PTP1B inhibition *in vitro* and controlled hyperglycemic index in *in vivo* experiments. In addition, PTP1B expression was also inhibited in diabetic treated mice with which was overexpressed in STZ-induced diabetic mice.

**FIGURE 6:**
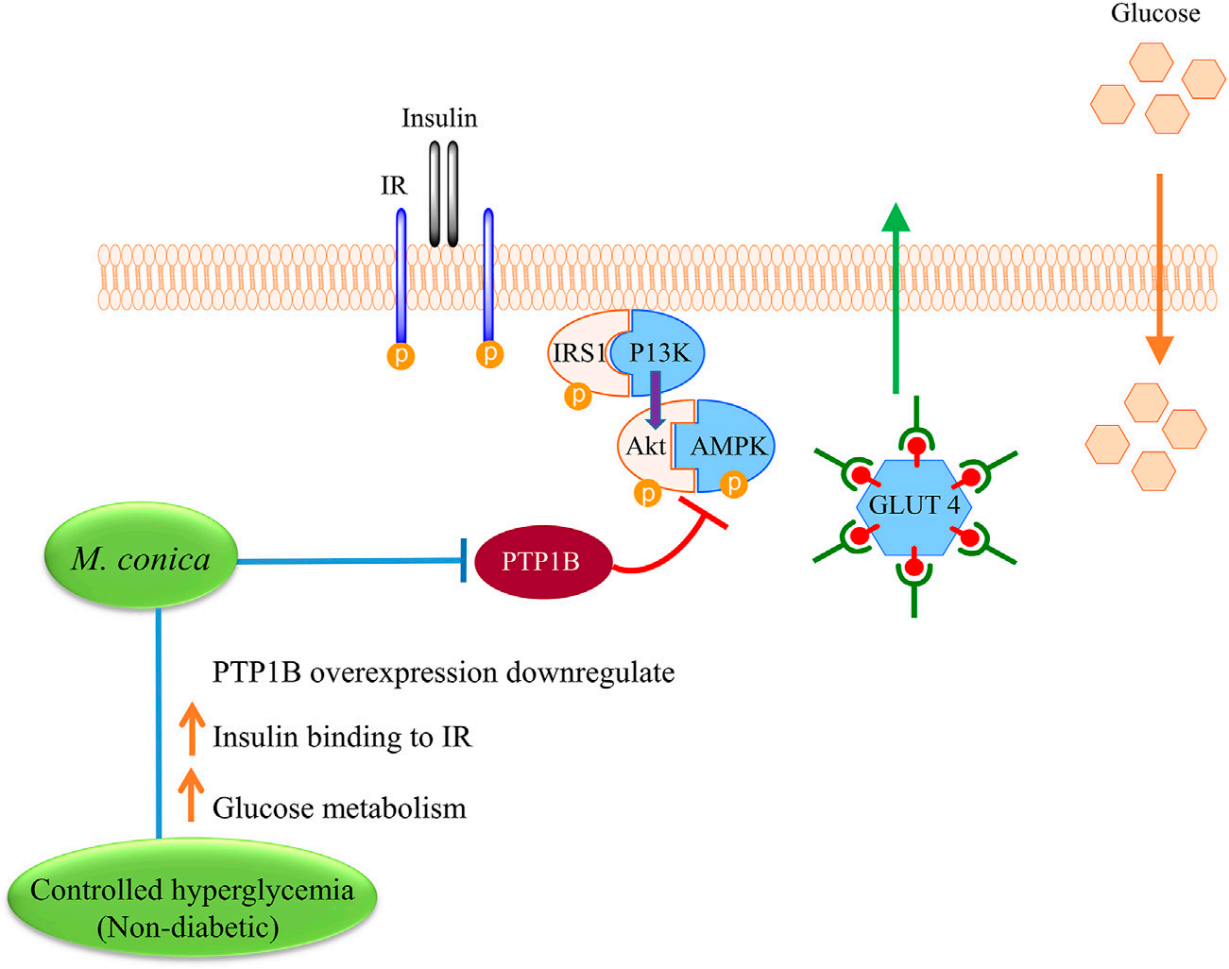
Proposed mechanism for exploring possible effect of *M. conica* on improving insulin binding to insulin receptor (IR) and increase glucose metabolism. *M. conica*, decrease the PTP1B expression and increase the phosphorylation of PTP1B targets in the insulin signaling pathways. This leads to increase the insulin sensitivity and reduce the blood glucose.

PTP1B is a reported negative regulator of the insulin signaling pathway as illustrated in Figure 6 (Ma et al., 2011; Zhou et al., 2021). The group studied the effect of PTP1B inhibitor CCF06240 on lipid metabolic abnormalities and insulin sensitivity *in vitro* and *in vivo*. The insulin-resistant mouse model was induced by a high-fat diet to study the lipid profile (Reuter et al., 2010). As a result, due to the presence of PTP1B inhibitor (CCF06240) insulin resistance was improved. TG, TC and body weight were also reduced. These results demonstrated that CCF06240 (PTP1B inhibitor) could increase insulin sensitivity through the regulation of the insulin signaling pathway.

Shrikrishna et al., 2000 investigated the cellular mechanism(s) of insulin resistance associated with non-insulin dependent diabetes mellitus (NIDDM) using skeletal muscles isolated from Goto-Kakizaki (GK) rats (genetic rat model for type II diabetes). It was noticed that as compared to control mice GK mice showed insulin stimulated insulin receptor autophosphorylation and insulin receptor substrate-1 tyrosine phosphorylation was prominently inhibited in GK skeletal muscles. This may be due to increased dephosphorylation by a protein tyrosine phosphatase (PTPase). It was noticed that PTPase 1B activity was increased in diabetic rats (GK rats). Therefore it was concluded that enhanced PTP1B activity leads to impaired glucose tolerance and enhanced insulin resistance (Dadke et al., 2000). Our results were found consistent with these studies as *M. conica* inhibited PTP1B activity at a concentration of low micromolar range. In addition, *in vivo* data also revealed decreased PTP1B expression in the liver by *M. conica* leading to a controlled hyperglycemic index which indicated that hyperglycemia in diabetic mice was due to overexpression of PTP1B which causes insulin resistance thus elevated levels of plasma glucose.

## Conclusion

Following conclusions can be drawn from this study: Firstly, *M. conica* which is an edible mushroom can be a potential source of anti-diabetic drug as it shows controlled hyperglycemic index and other related parameters in *vivo* experiments. Secondly, the proposed mechanism by which *M. conica* controlled hyperglycemic index is an alteration of PTP1B protein expression in liver and pancreas which was found to be overexpressed in case of diabetic mice liver and decreased in diabetic mice pancreas. However, in *M. conica-*treated groups controlled expressions were found for PTP1B. In addition, the present study also revealed the presence of possible phytochemicals by LC-MS. *M. Conica* is a potential source of diabetes mellitus treatment involved in reducing elevated blood glucose during diabetes, which has been shown to be further studied by oral hypoglycemic therapy. Furthermore, in future, the chemical composition should be described in detail which could expand our understanding of the possible mechanism of *M. conica* in diabetes.

## Data Availability

The original contributions presented in the study are included in the article/Supplementary Material, further inquiries can be directed to the corresponding author.
