# Supplementary material for: Evaluation of the Hypoglycemic Activity of Morchella conica by Targeting Protein Tyrosine Phosphatase 1B
Source: Front Pharmacol. 2021 May 14;12:661803. doi: 10.3389/fphar.2021.661803 (PMC8173442; doi:10.3389/fphar.2021.661803)
Supplement: Supplementary file 1 [file DataSheet1.PDF]

LC-MS analysis and *in-vivo* anti-diabetic potential of *Morchella conica* by targeting protein tyrosine phosphatase 1B

Naeema Begum[1], Abdul Nasir[1,2], Nargis Jamilla[3], Mohib Shah[4], Noor Shad Bibi[4], Akif Khurshid[1], Taj Muhammad[1], Asma Ahmad[5], Zille Huma[6], Atif Ali Khan Khalil[7], Saira Farman[1], Zahida Parveen[1*]

[1]Department of Biochemistry, Abdul Wali Khan University, Mardan, Khyber Pakhtunkhwa, Pakistan

[2]Department of Molecular Science and Technology, Ajou University, Suwon, Republic of Korea

[3]Department of Chemistry, Shaheed Benazir Women University of Science and technology Peshawar, Khyber Pakhtunkhwa, Pakistan

[4]Department of Botany, Abdul Wali Khan University, Mardan, Khyber Pakhtunkhwa, Pakistan

[5]Institute of Molecular Biology and Biotechnology, The University of Lahore, Lahore, Pakistan

[6]Department of Botany, University of Peshawar, Khyber Pakhtunkhwa, Pakistan.

[7]Department of Biological Sciences, National University of Medical Sciences, Rawalpindi 46000, Pakistan.

*** Correspondence:**

Zahida Parveen, PhD
Department of Biochemistry, Abdul Wali Khan University, Mardan, Khyber Pakhtunkhwa, Pakistan
Email: zahida@awkum.edu.pk

Supplementary

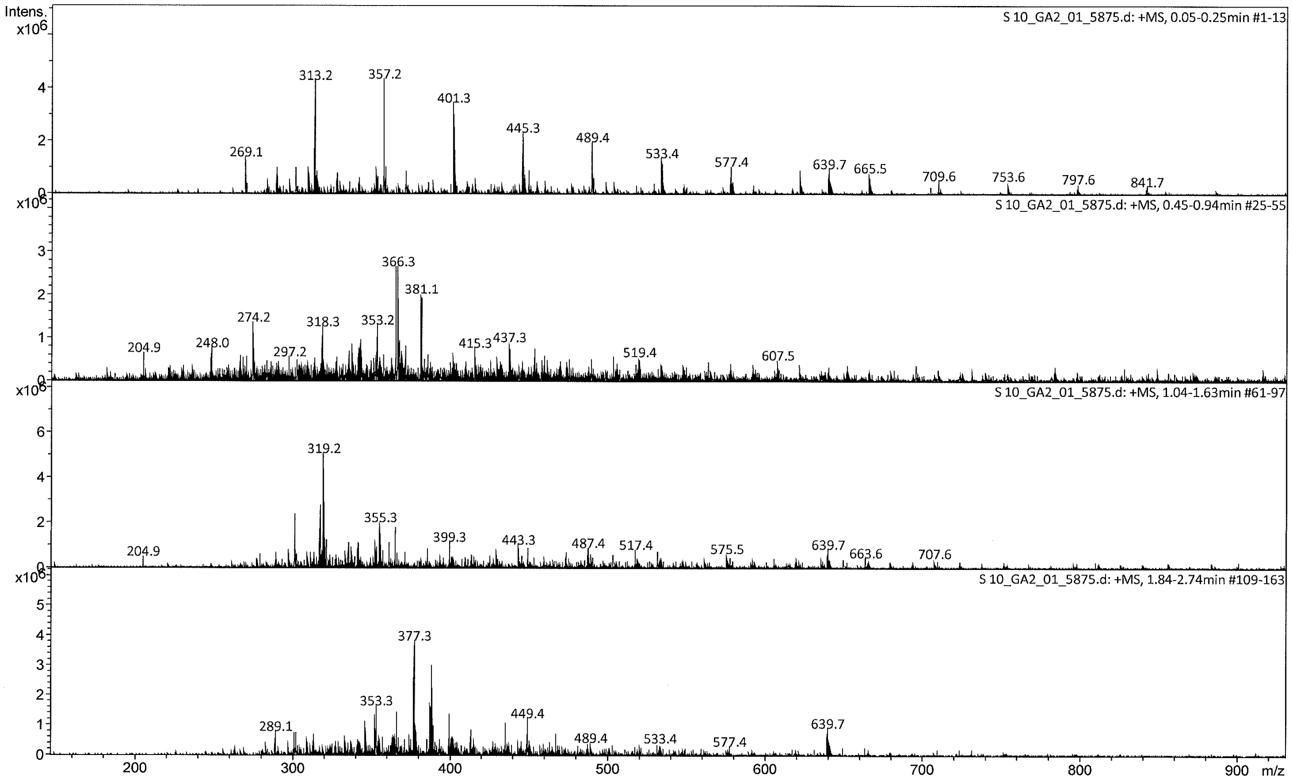

Figure S1.1. ESI mass spectrum (+MS) of S10 at 0.05-0.25, 0.45-0.94, 1.04-1.63, and 1.84-2.74 min

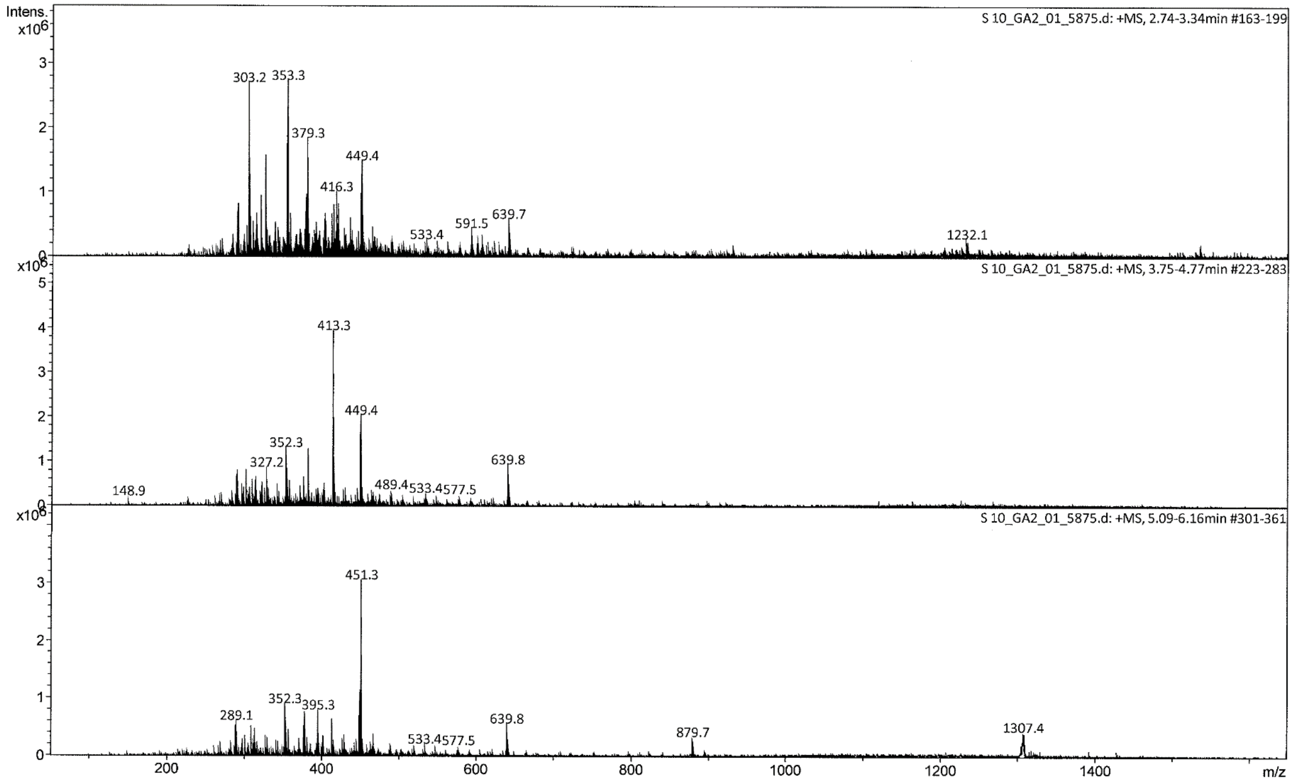

Figure S1.2. ESI mass spectrum (+MS) of S10 at 2.74-3.34, 3.75-4.77, and 5.09-6.16 min

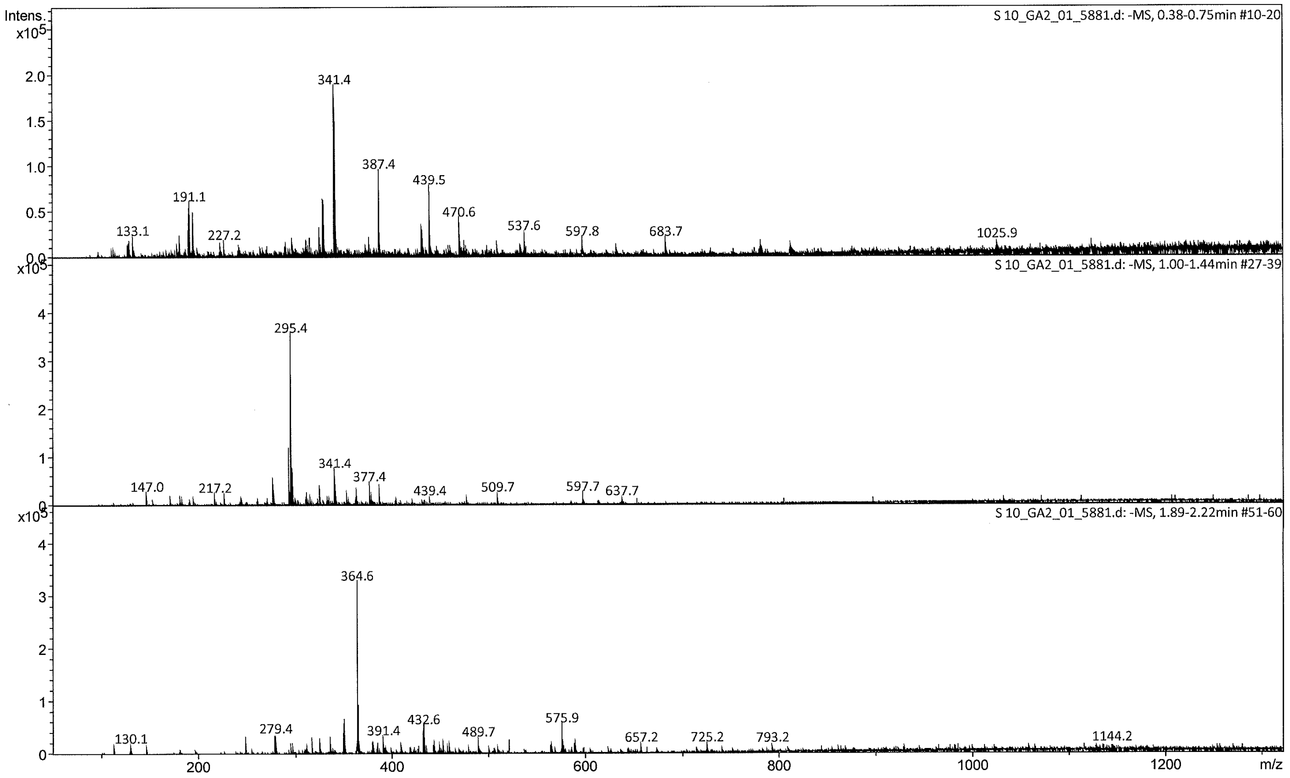

Figure S1.3. ESI mass spectrum (-MS) of S10 at 0.38-0.75, 1.00-1.44, and 1.89-2.22 min

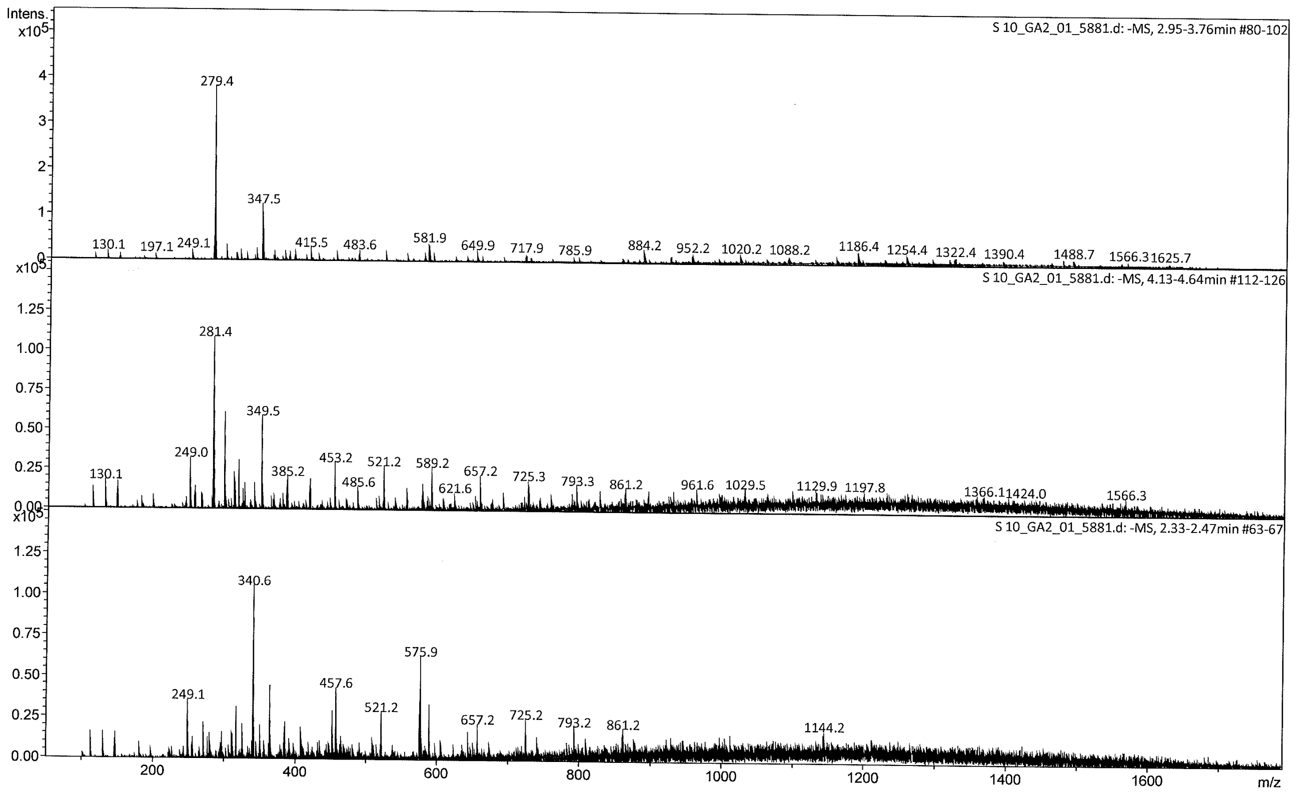

Figure S1.4. ESI mass spectrum (-MS) of S10 at 2.95-3.76, 4.13-4.64, and 2.33-2.47 min
